# Supplementary material for: Nitrogen availability regulates topsoil carbon dynamics after permafrost thaw by altering microbial metabolic efficiency
Source: Nat Commun. 2018 Sep 27;9:3951. doi: 10.1038/s41467-018-06232-y (PMC6160441; doi:10.1038/s41467-018-06232-y)
Supplement: Supplementary file 1 — Supplementary Information [file 41467_2018_6232_MOESM1_ESM.pdf]

Supplementary Information

Nitrogen availability regulates topsoil carbon dynamics after permafrost thaw by altering microbial metabolic efficiency

Chen et al.

Supplementary Note 1: Different interaction effects in gradient experiment and N addition experiment

In the gradient experiment, there was no significant glucose (G) × thaw sequence (time) interaction on qCO_2 (P = 0.14, Fig. 4c), suggesting that the significant enhancement on qCO_2 induced by glucose addition did not vary with thaw sequence. In contrast, a significant interaction between glucose and N addition (P = 0.04, Supplementary Fig. 8) on qCO_2 was observed in the subsequent N addition experiment. Specifically, the magnitude of qCO_2 promoted by glucose addition decreased with increasing N availability (Supplementary Fig. 8). Similar contradictions between the two experiments were also observed for BG (Supplementary Fig. 7a, Supplementary Fig. 9a) and LAP activities (Fig. 4a, Supplementary Fig. 9c). These discrepancies could be attributed to the fact that not only N availability but also many other soil factors varied along the thaw sequence (Supplementary Table 1). These confounding factors together with N availability co-affected the pattern in the qCO_2, BG and LAP activities along the thaw sequence. However, in the N addition experiment, these confounding factors were held constant before N supply. It is thus possible to observe different effects of "time" and "nitrogen" treatment on these response variables.

Supplementary Note 2: Effects of enzyme activity on priming effect along the thaw sequence

Enzyme activity has been assumed to be the mechanism responsible for the variations of priming effect along N availability[1,2], because it is directly involved in the biodegradation of SOM as a catalyst. In previous studies, the significant correlation between SOM-derived CO_2 flux and enzyme activity was considered as the evidence of the role of enzyme activity in priming effect [1,3,4]. Similarly, a significant correlation between soil C release and enzyme activity was also observed in our study (Supplementary Fig. 7d). However, unlike previous results obtained in low fertility soils[1,4,5], we found that N-acquiring enzyme (*i.e.*, LAP) rather than C-acquiring enzyme (*e.g.*, BG and POX) contributed to the enhanced SOM-derived CO_2 release after glucose addition (Fig. 4a, Supplementary Fig. 7d). This discrepancy could be attributed to the relatively weaker microbial energy limitation in our swamp meadow soil than that in other soils (indicative of higher soil organic C concentration: 18.6% *vs.* 0.9~2.8%)[1,4,5]. This relatively weak energy limitation in our study site could be partly relieved after glucose addition, which in turn aggravates the N limitation (Supplementary Fig. 4b; indicative of higher C:N imbalance after glucose addition) and thus promotes the N-acquiring enzyme activity. This assumption was also supported by the non-significant response of C-acquiring enzyme after labile C addition in a tundra soil characterized by high organic C content (48%) and low energy limitation[21]. Additionally, given that the enzyme activity usually varies with time, different sampling time during incubation may also partly contribute to

the discrepancy among studies. Nevertheless, it should be mentioned that the correlation between LAP and SOM-derived CO_2 release only reflects the effects of enzyme on SOM decomposition, rather than on the priming effect (*i.e.*, the magnitude of the change in SOM decomposition after labile C addition). The lack of correlation between priming effect and LAP (Fig. 4d) further demonstrated that enzyme activity was not the major mechanism responsible for the variations of priming effect along the thaw sequence.

Supplementary Table 1. Soil properties in the top 15 cm along a permafrost thawing sequence on the Tibetan Plateau.

	Control	1 year	10 years	16 years
Moisture (wt %)	202.0±8.3 a	175.0±6.6 b	187.5±5.0 ab	154.8±9.5 b
SOC (g kg^{-1})	186.6±4.8 a	178.9±4.7 a	183.7±3.1 a	152.1±10.5 b
SOCD (kg C m^{-3})	54.1± 1.4 a	55.5±1.5 a	55.1±0.9 a	48.7±3.3 a
STN (g kg^{-1})	15.2±0.4 a	14.8±0.3 ab	15.3±0.3 a	12.6±0.9 b
STND (kg N m^{-3})	4.4±0.1 a	4.6±0.1 a	4.6±0.1 a	4.0±0.3 a
$R_{C:N}$	12.3±0.06 a	12.1±0.05 a	12.0±0.06 a	12.0±0.07 a
DOC (mg kg^{-1})	161.7±5.9 a	161.5±6.9 a	195.9±7.3 a	178.2±10.8 a
TDN (mg kg^{-1})	33.1±1.5 c	47.5±3.0 a	43.1±2.3 ab	37.8±1.8 bc
Clay (%)	2.1±0.04 a	2.1±0.05 a	2.1±0.03 a	2.0±0.06 a
Silt (%)	53.1±0.6 a	53.6±1.0 a	53.4±0.7 a	49.5±1.3 a
$B_{C:N}$	17.5±0.4 a	17.1±1.0 a	15.5±0.6 ab	14.6±0.7 b
$R_{TOC:TDN}/B_{C:N}$	0.28±0.007 b	0.20±0.009 c	0.29±0.007 a	0.32±0.016 a

Mean values±SE (n = 5) are displayed. SOC, soil organic carbon; SOCD, soil organic carbon density; STN, soil total N; STND, soil total N density; $R_{C:N}$, soil C:N ratio; DOC, dissolved organic carbon; TDN, total dissolved nitrogen; $B_{C:N}$, microbial biomass C:N ratio; $R_{TOC:TDN}/B_{C:N}$, the ratio of resource C:N ($R_{DOC:TDN}$, ratio of soil DOC to TDN) normalized to microbial biomass C:N ($B_{C:N}$)[6].

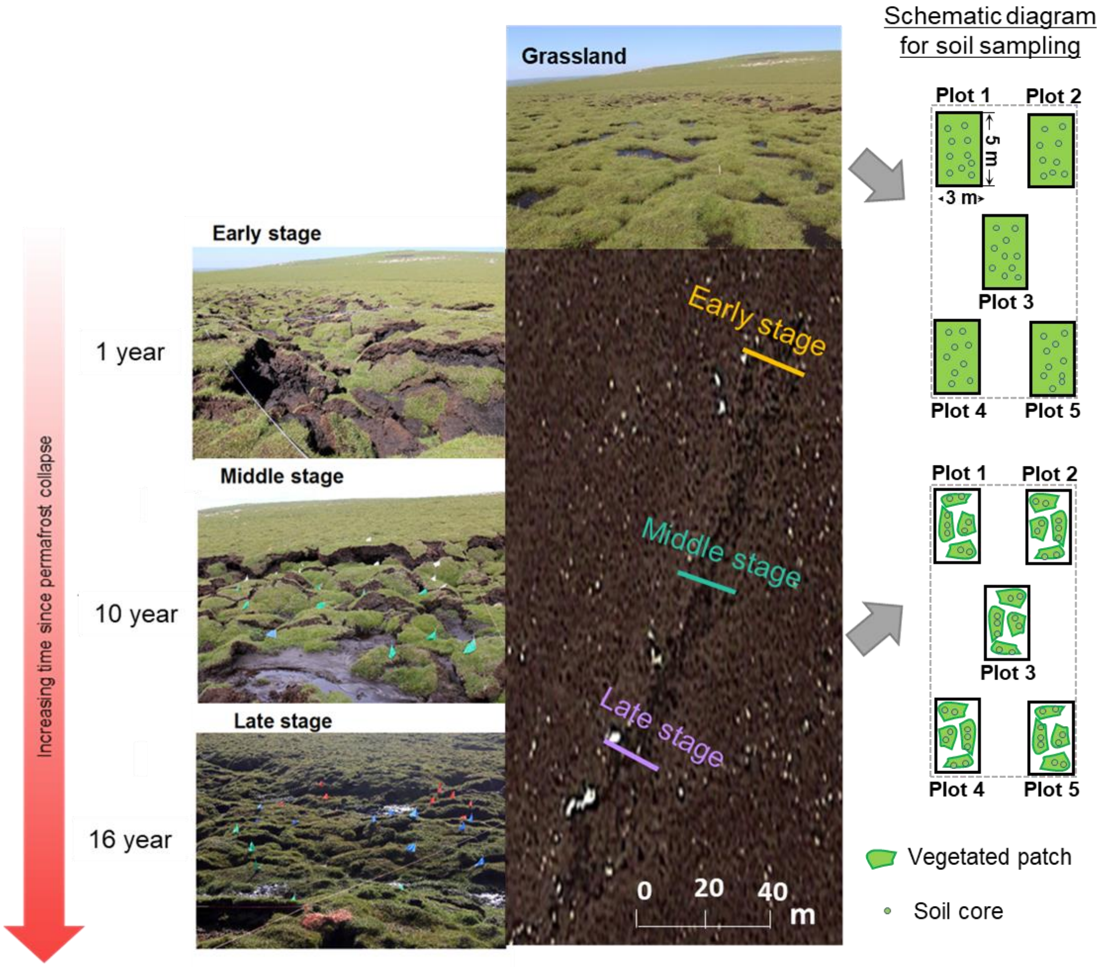

Supplementary Figure 1. Landscapes of different collapse stages within a typical thermo-erosion gully on the Tibetan Plateau and the sampling schematic diagram. The years since permafrost collapse for 1, 10 and 16 years were named as early-, middle- and late-stage of permafrost collapse, respectively.

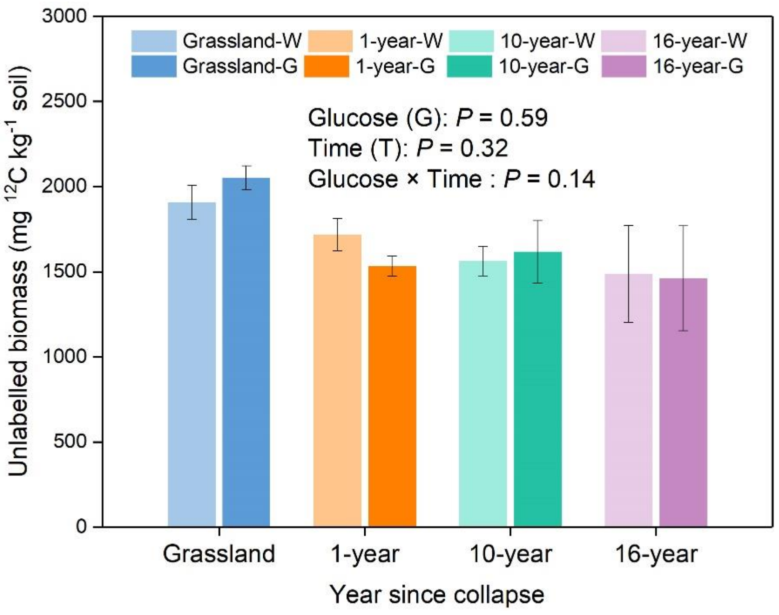

Supplementary Figure 2. Unlabelled microbial biomass C after incubation in control and glucose amended soils across different collapse stages. Data represent means ± SE (standard error). W and G in legends refer to water and glucose addition treatment, respectively.

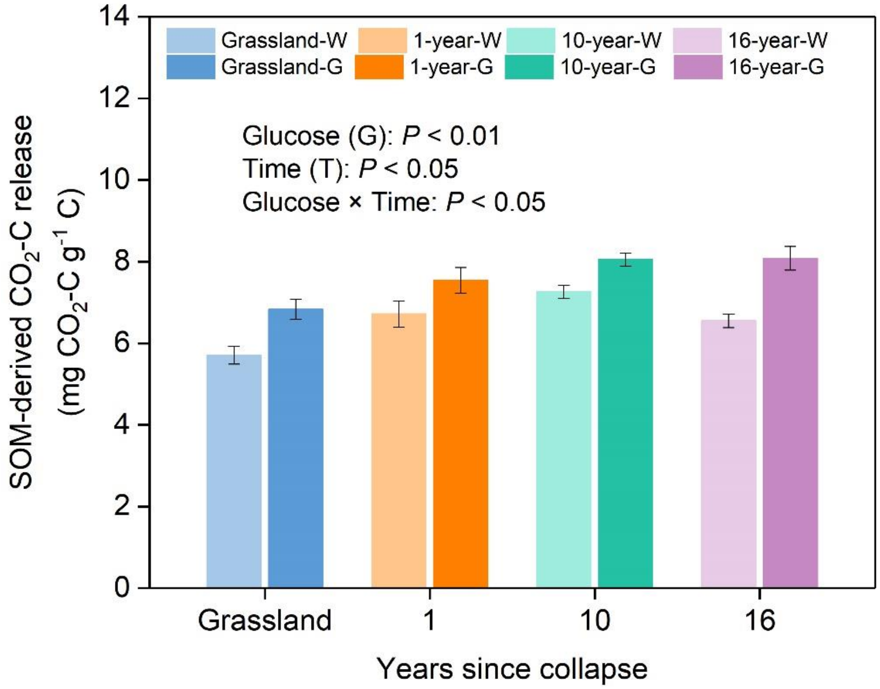

Supplementary Figure 3. Cumulative SOM-derived CO_2-C release in control and glucose amended soils across different collapse stages. Data represent means ± SE (standard error).

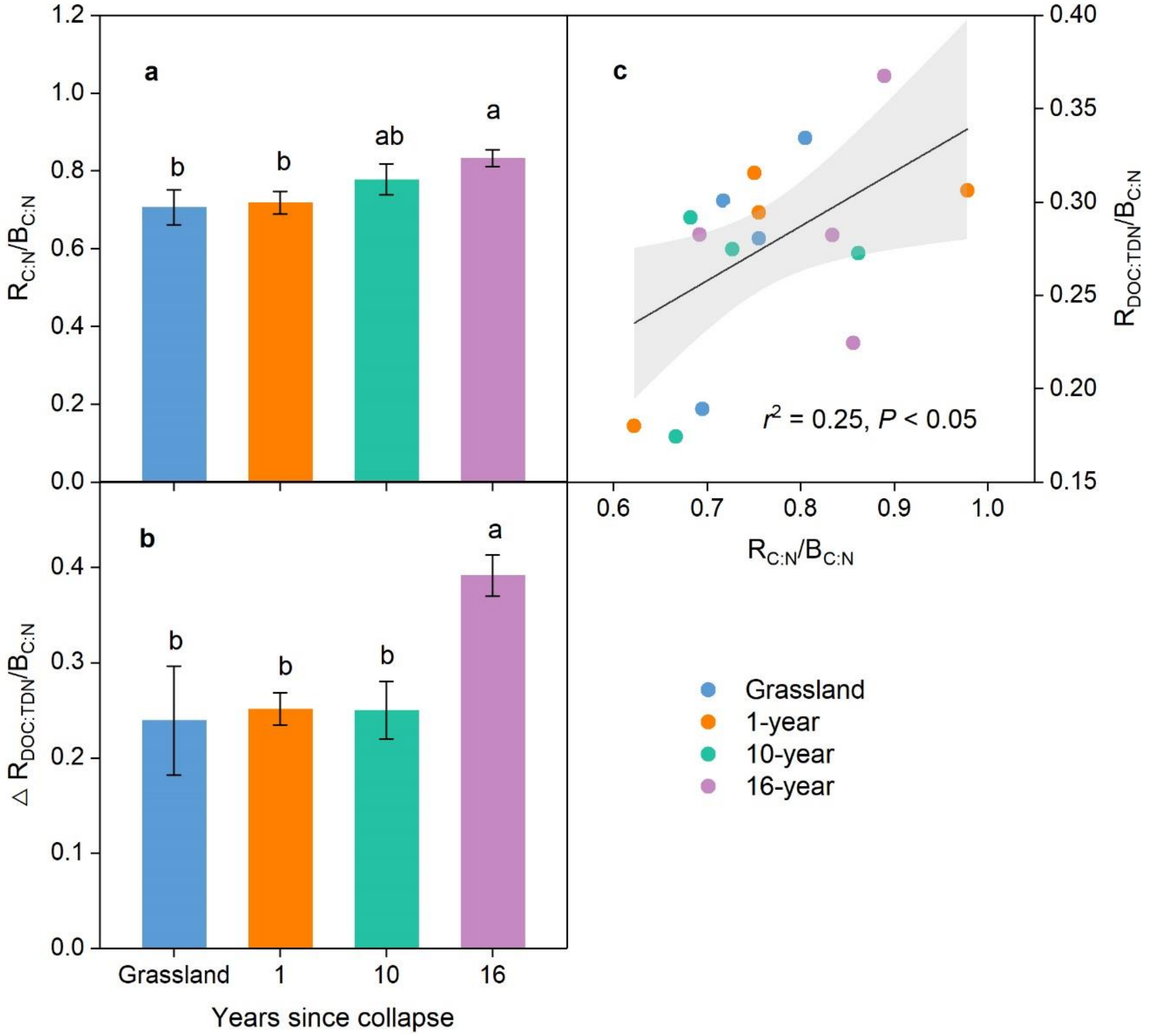

Supplementary Figure 4. Variations of stoichiometric imbalance between resources and microorganism ($R_{C:N}/B_{C:N}$) along the thaw sequence. (**a**) total form and (**b**) changes in the labile form of stoichiometric imbalance ($R_{TOC:TDN}/B_{C:N}$) after glucose addition. (**c**) Relationship between the $R_{C:N}/B_{C:N}$ and $R_{TOC:TDN}/B_{C:N}$. Data represent means ±SE (standard error). Significant differences are denoted by different letters ($P < 0.05$).

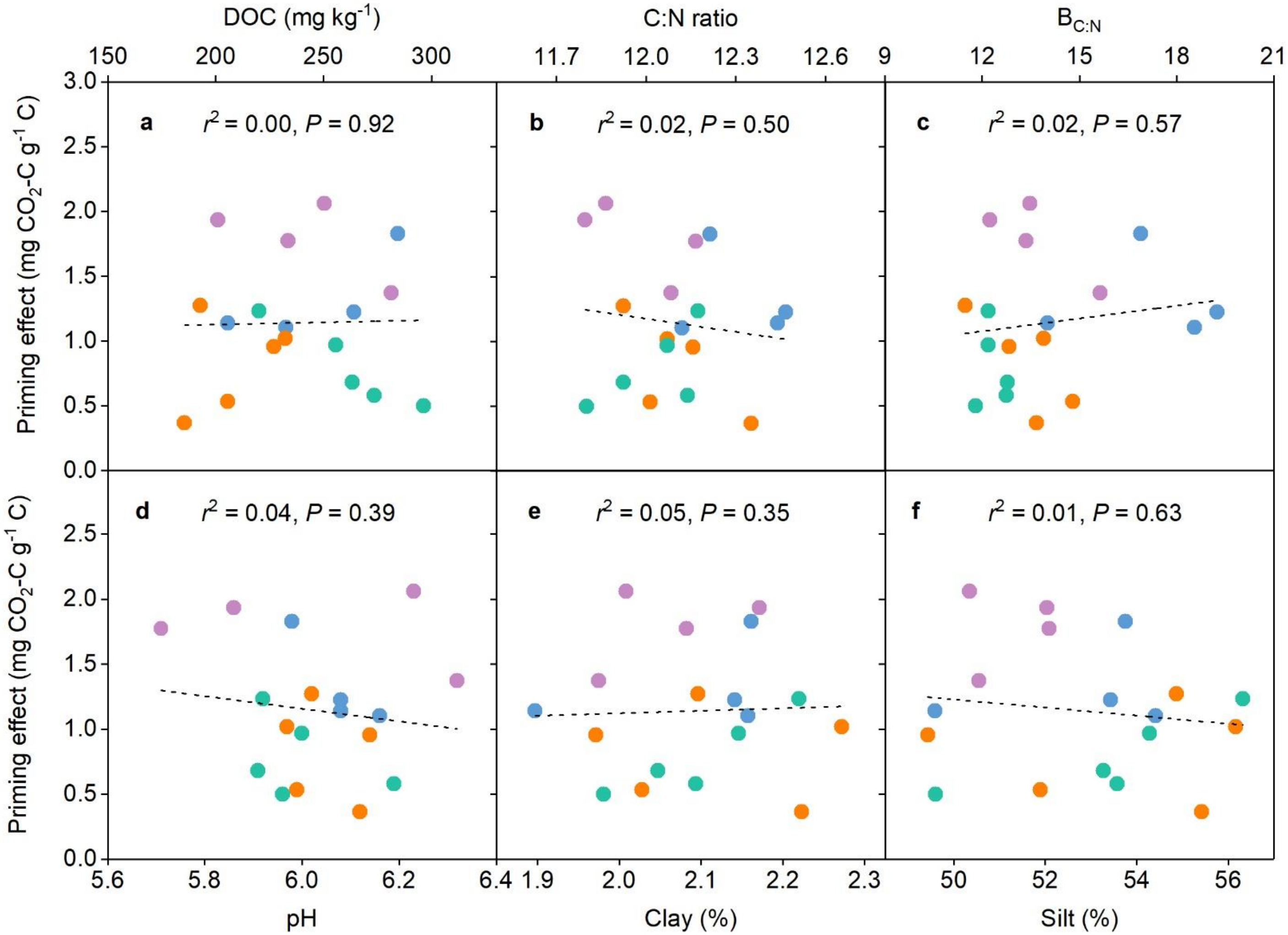

Supplementary Figure 5. **Relationships between the priming effect and various soil parameters**. (**a**) dissolved organic carbon (DOC), (**b**) C:N ratio, (**c**) microbial biomass C:N ratio ($B_{C:N}$), (**d**) pH, (**e**) clay and (**f**) silt content in the top 15 cm under the glucose treatment.

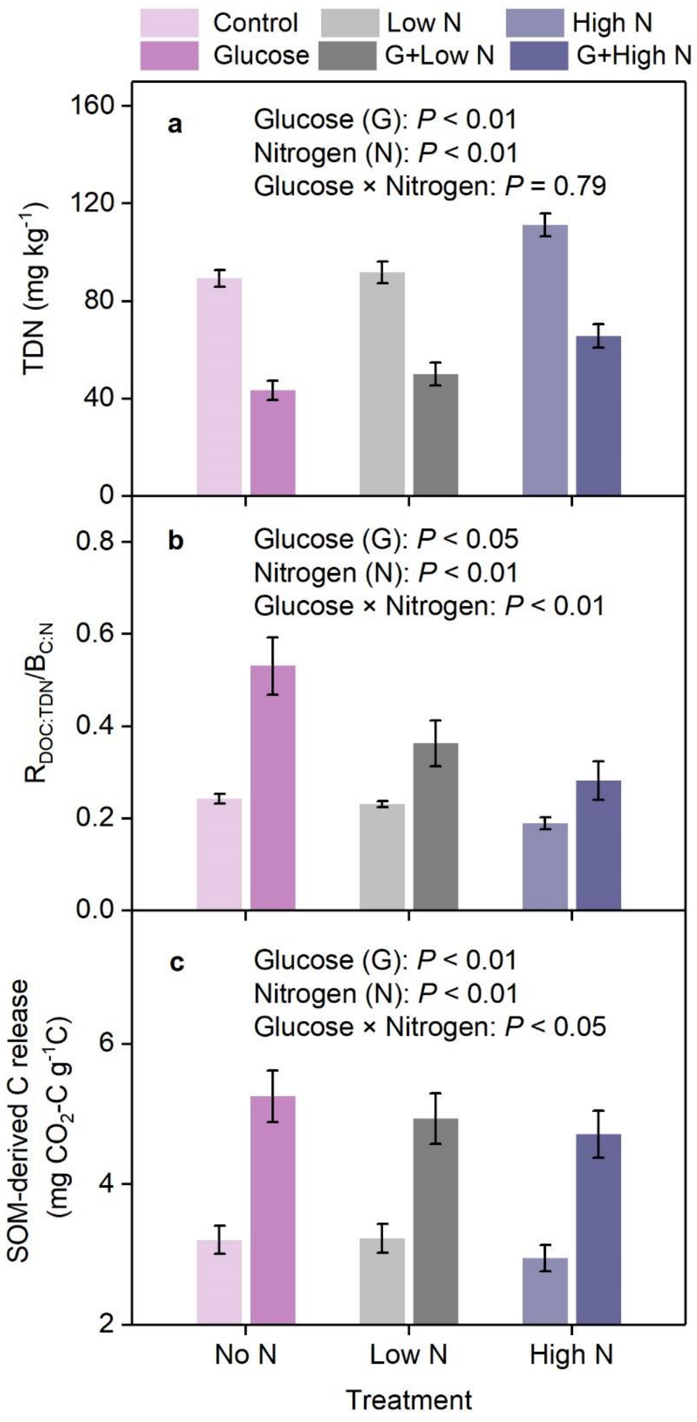

Supplementary Figure 6. Key N parameters and soil CO_2-C release in N addition experiment. (a) Soil total dissolved nitrogen (TDN) concentration, (**b**) C:N imbalance ($R_{DOC:TDN}/B_{C:N}$) and (**c**) SOM-derived C release under glucose and N addition treatments. Data represent means ± SE (standard error).

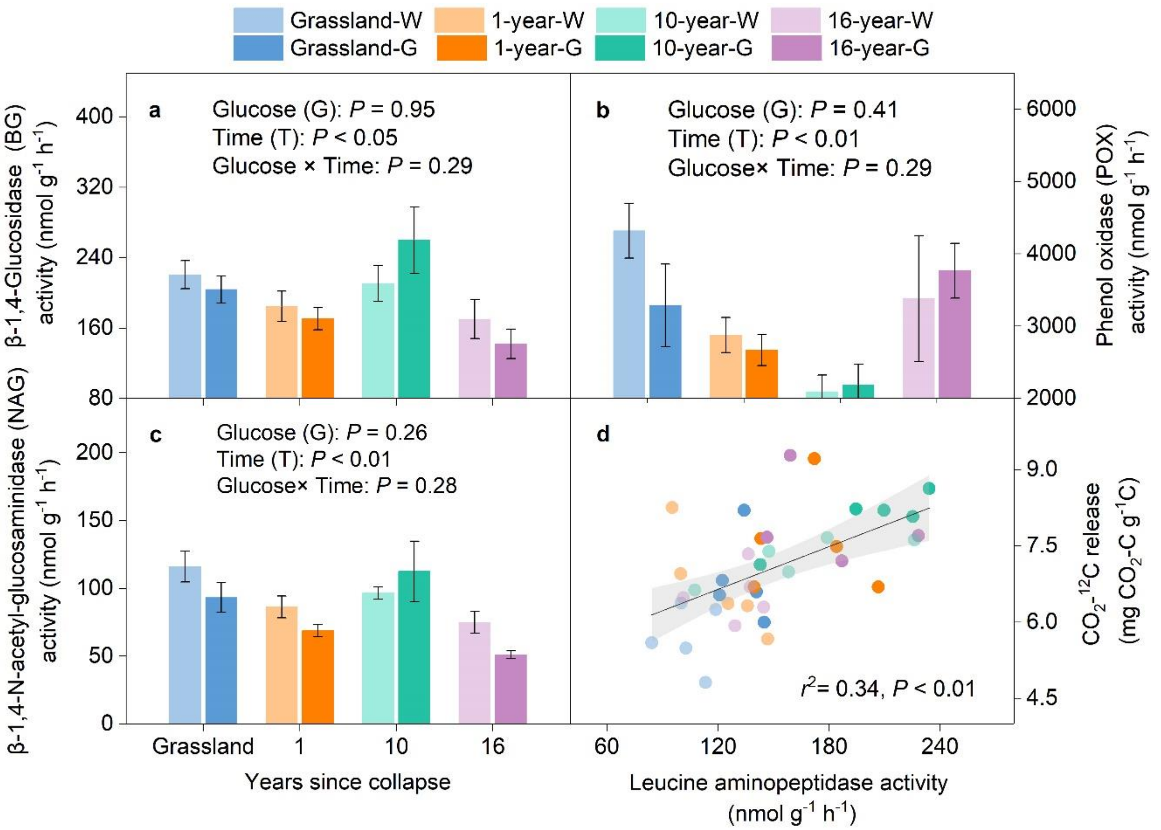

Supplementary Figure 7. Extracellular enzyme activities under water and glucose addition treatments along the thaw sequence. (**a**) *β*-1,4-glucosidase (BG), (**b**) phenol oxidase (POX) and (**c**) *β*-1,4-N-acetylglucosaminidase (NAG). (**d**) The relationship between SOM-derived CO_2-C release and leucine aminopeptidase (LAP) activity along the thaw sequence. Data represent means ± SE (standard error).

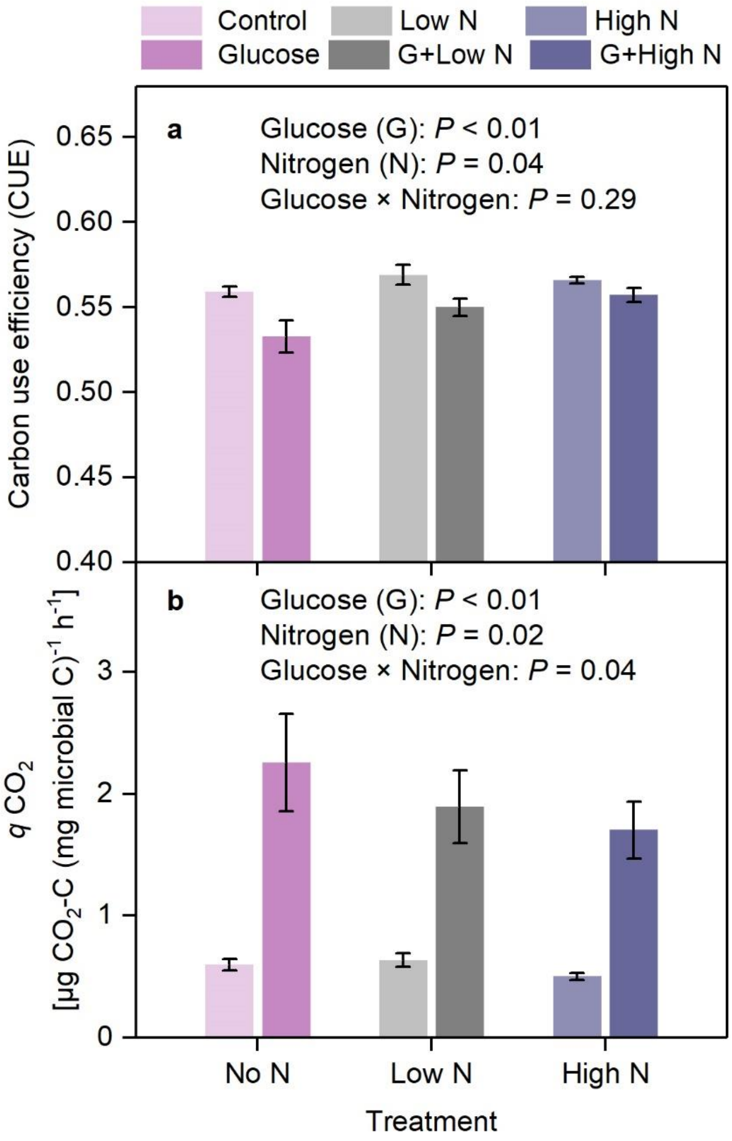

Supplementary Figure 8. Microbial metabolic efficiency under glucose and N addition treatments. (**a**) C use efficiency (CUE) and (**b**) qCO_2. Data represent means ± SE (standard error).

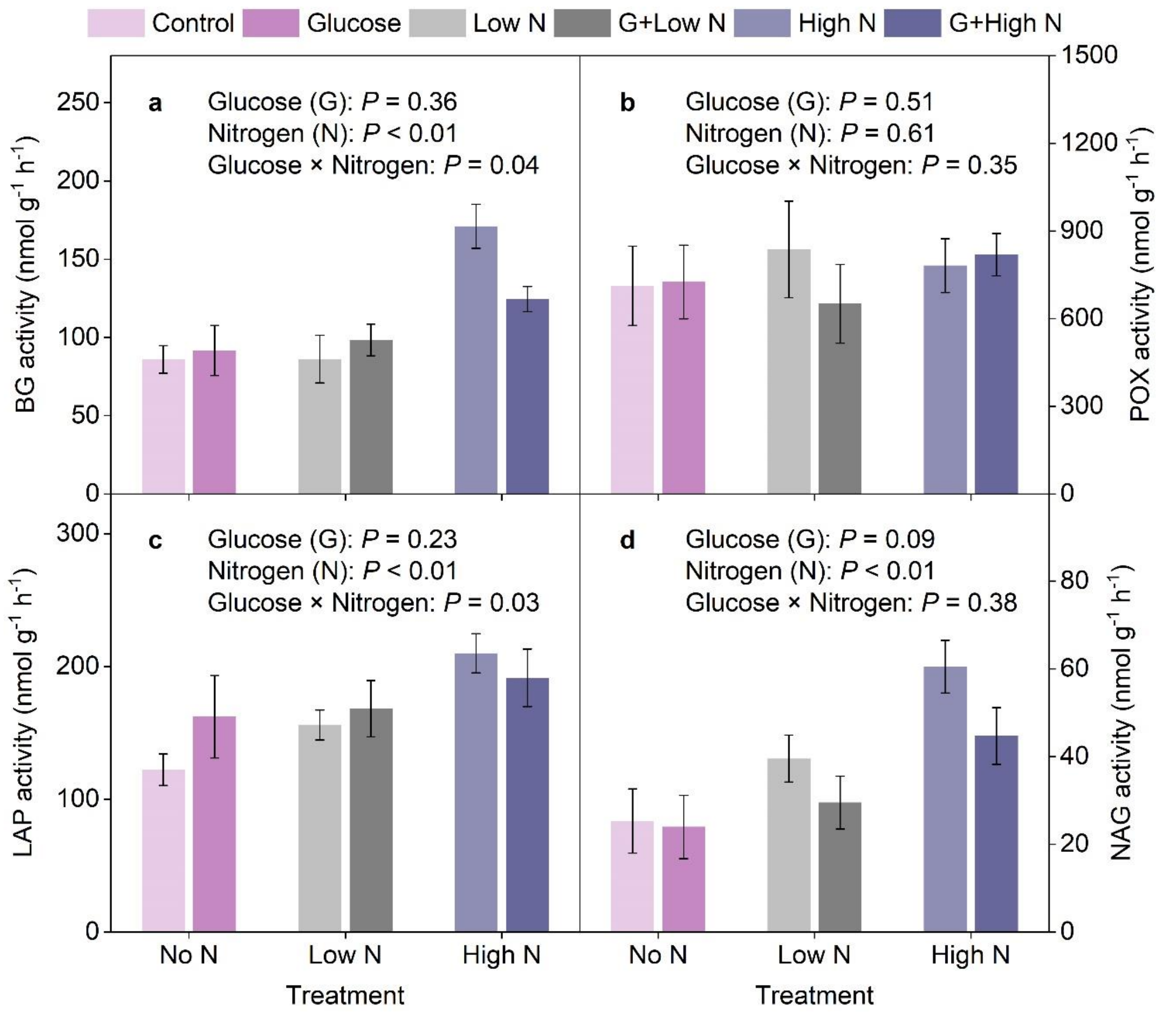

Supplementary Figure 9. Extracellular enzyme activities under glucose and N addition treatments. (**a**) *β*-1,4-glucosidase (BG), (**b**) phenol oxidase (POX), (**c**) leucine aminopeptidase (LAP), and (**d**) *β*-1,4-N-acetylglucosaminidase (NAG). Data represent means ± SE (standard error)

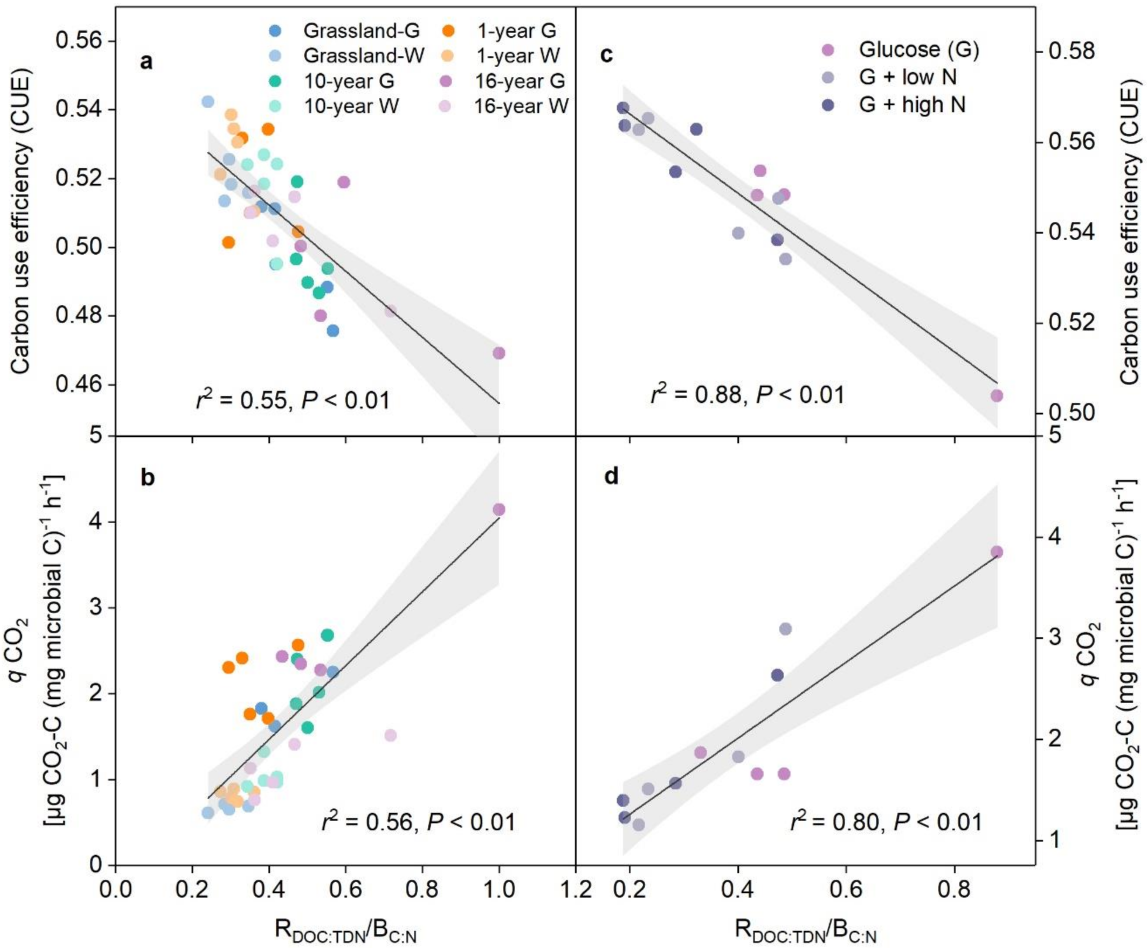

Supplementary Figure 10. Relationship between microbial metabolic efficiency and C:N imbalance ($\mathbf{R_{DOC:TDN}/B_{C:N}}$). (**a, c**) C use efficiency (CUE) and (**b, d**) metabolic quotients (qCO_2) in the gradient experiment (**a, b**) and N addition experiment (**c, d**).

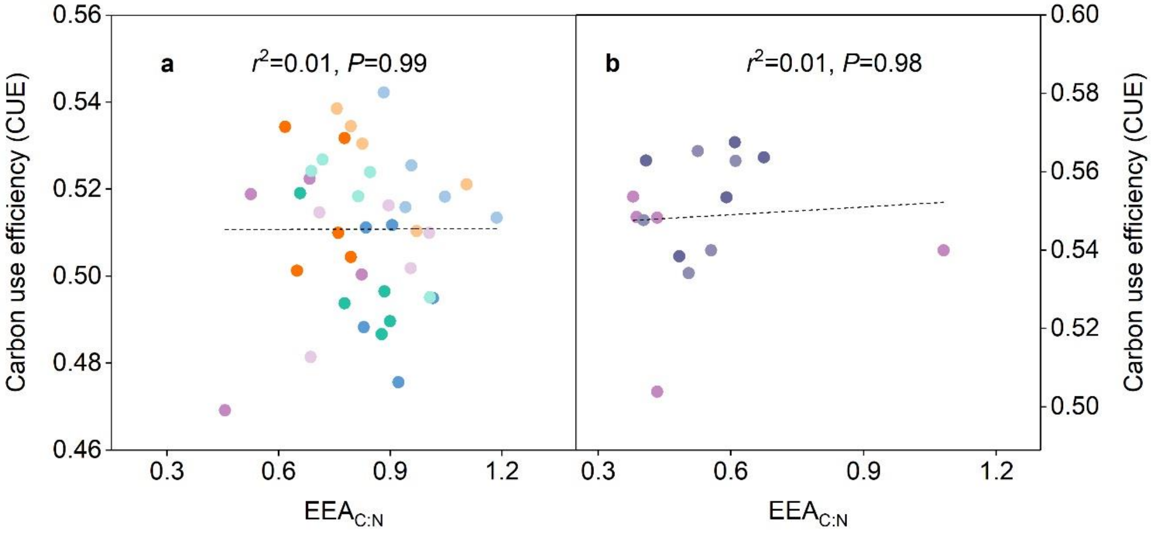

Supplementary Figure 11. Relationship between microbial C use efficiency (CUE) and enzyme stoichiometric ratio (EEA$_{C:N}$). (**a**) gradient experiment, (**b**) N addition experiment.

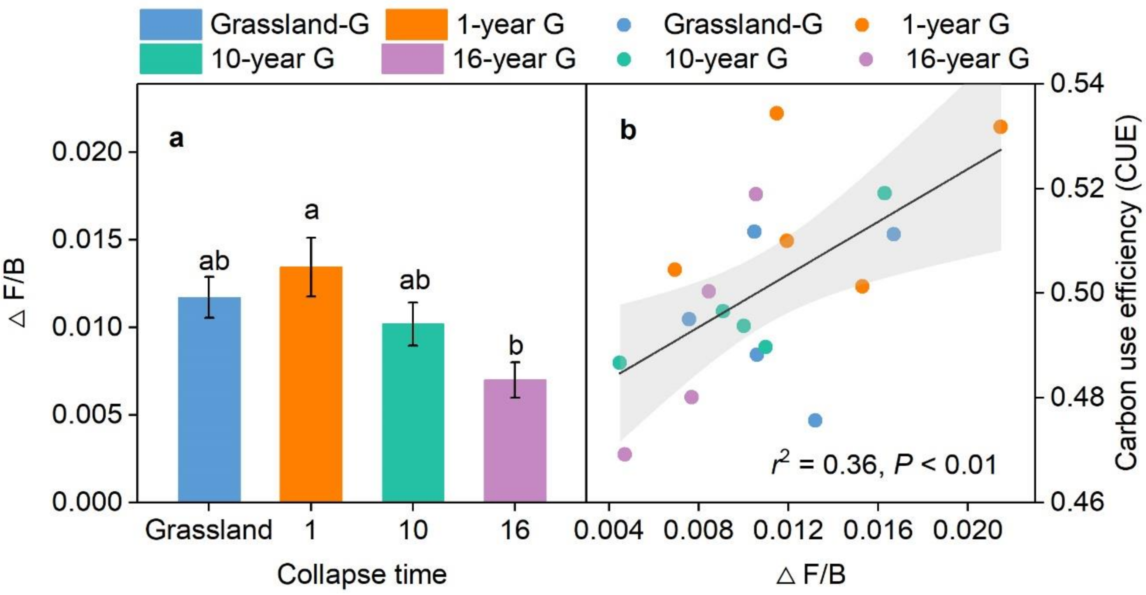

Supplementary Figure 12. Change in fungi/bacterial (F/B) ratio after glucose addition and its relationship with microbial C use efficiency (CUE). (**a**) changes in F/B ratio along the thaw sequence, (**b**) relationship between the changes and CUE along the thaw sequence. Data represent means ± SE (standard error).

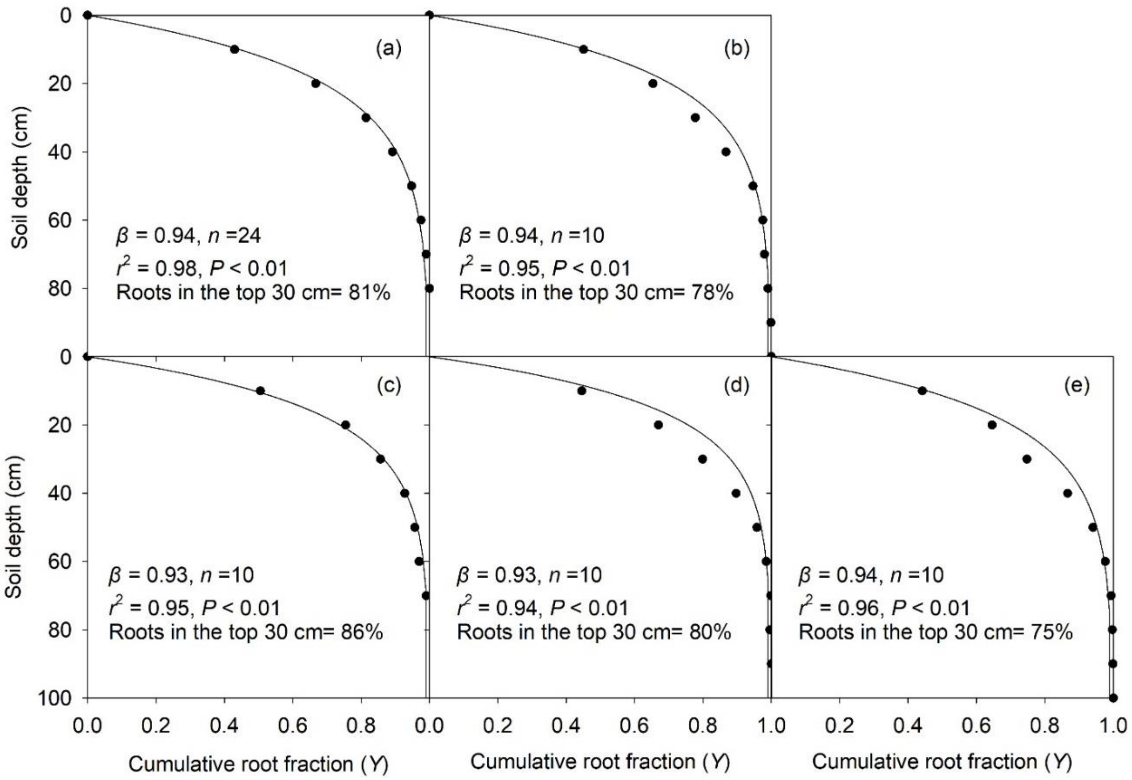

Supplementary Figure 13. Vertical distributions of roots in the swamp meadow. (**a**) the swamp meadow across the Tibetan Plateau, (**b-e**) the swamp meadow in this study with different thaw stages: (**b**) undisturbed grassland site, (**c**) early-stage, (**d**) middle-stage, (**e**) late-stage of the permafrost collapse. The vertical distribution of roots was fitted by the function proposed by Gale and Grigal [7]. This was characterized as $Y = 1-\beta^{d}$, where Y is a cumulative percentage of root biomass from the soil surface to depth d (cm), and β is the fitted parameter. The data for root distributions in the whole swamp meadow across the plateau was obtained from the regional survey in 2005[8].

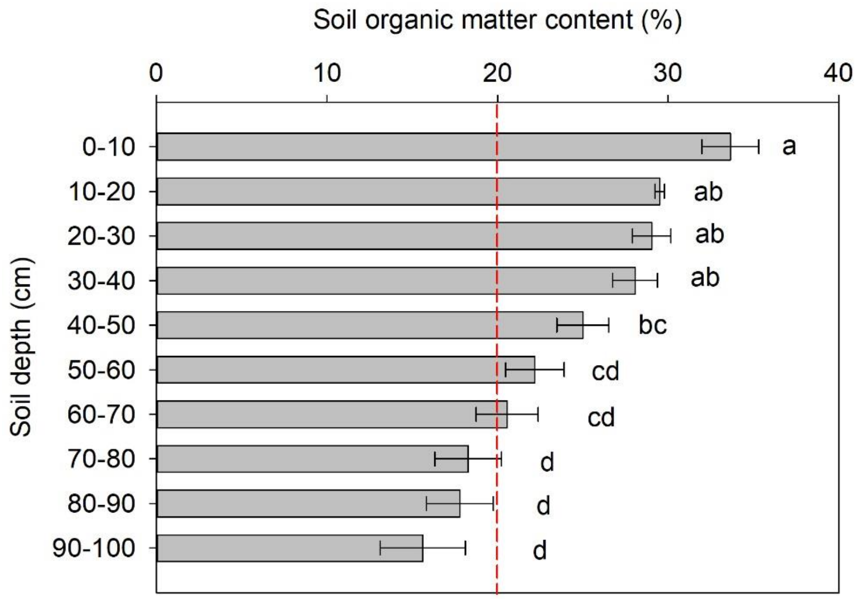

Supplementary Figure 14. **Vertical distributions of soil organic matter (SOM) in our study site**. The SOM content was estimated from the soil organic C concentration using the conversion factor of 1.724. Data represent means ± SE (standard error). Mean values with different letters indicate significant differences among soil depths ($P < 0.05$).

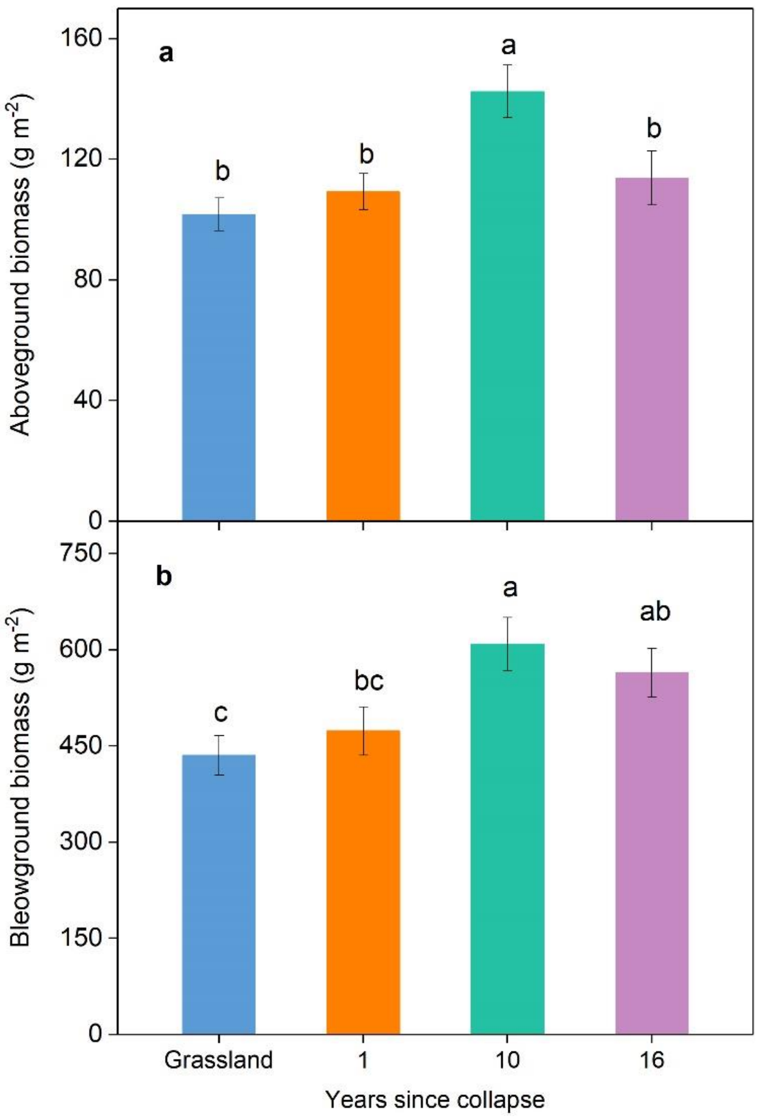

Supplementary Figure 15. **Changes in vegetation biomass along the thaw sequence.** (**a**) above- and (**b**) belowground plant biomass.

Supplementary References

1. Chen, R. *et al.* Soil C and N availability determine the priming effect: microbial N mining and stoichiometric decomposition theories. *Glob. Change Biol.* **20**, 2356-2367 (2014).
2. Carney, K. M., Hungate, B. A., Drake, B. G. & Megonigal, J. P. Altered soil microbial community at elevated CO_2 leads to loss of soil carbon. *Proc. Natl Acad. Sci. USA* **104**, 4990-4995 (2007).
3. Zhu, B., Gutknecht, J. L. M., Herman, D. J., Keck, D. C., Firestone, M. K. & Cheng, W. Rhizosphere priming effects on soil carbon and nitrogen mineralization. *Soil Biol. Biochem.* **76**, 183-192 (2014).
4. Zhu, B. & Cheng, W. Rhizosphere priming effect increases the temperature sensitivity of soil organic matter decomposition. *Glob. Change Biol.* **17**, 2172-2183 (2011).
5. Nottingham, A. T., Turner, B. L., Chamberlain, P. M., Stott, A. W. & Tanner, E. V. J. Priming and microbial nutrient limitation in lowland tropical forest soils of contrasting fertility. *Biogeochemistry* **111**, 219-237 (2012).
6. Mooshammer, M., Wanek, W., Zechmeister-Boltenstern, S. & Richter, A. Stoichiometric imbalances between terrestrial decomposer communities and their resources: mechanisms and implications of microbial adaptations to their resources. *Front. Microbiol.* **5**, 22 (2014).
7. Gale, M. R. & Grigal, D. F. Vertical root distributions of northern tree species in relation to successional status. *Can. J. For. Res.* **17**, 829-834 (1987).
8. Yang, Y., Fang, J., Ji, C. & Han, W. Above- and belowground biomass allocation in Tibetan grasslands. *J. Veg. Sci.* **20**, 177-184 (2009).
